# Supplementary material for: Unveiling hidden threats: Polycyclic aromatic hydrocarbons pollution in the glacial waters of the Meili Snow Mountains in the southeastern Tibetan Plateau
Source: PLoS One. 2025 Oct 16;20(10):e0334592. doi: 10.1371/journal.pone.0334592 (PMC12530526; doi:10.1371/journal.pone.0334592)
Supplement: S1 Fig — Data sources: Rivers and glaciers are extracted from Landsat 8 imagery, DEM (elevation) based on Advanced Spaceborne Thermal Emission and Reflection Radiometer (ASTER), was obtained from NASA (https://www.earthdata.nasa.gov/). All sources are in the public domain and not copyrighted. (DOCX) [file pone.0334592.s001.docx]

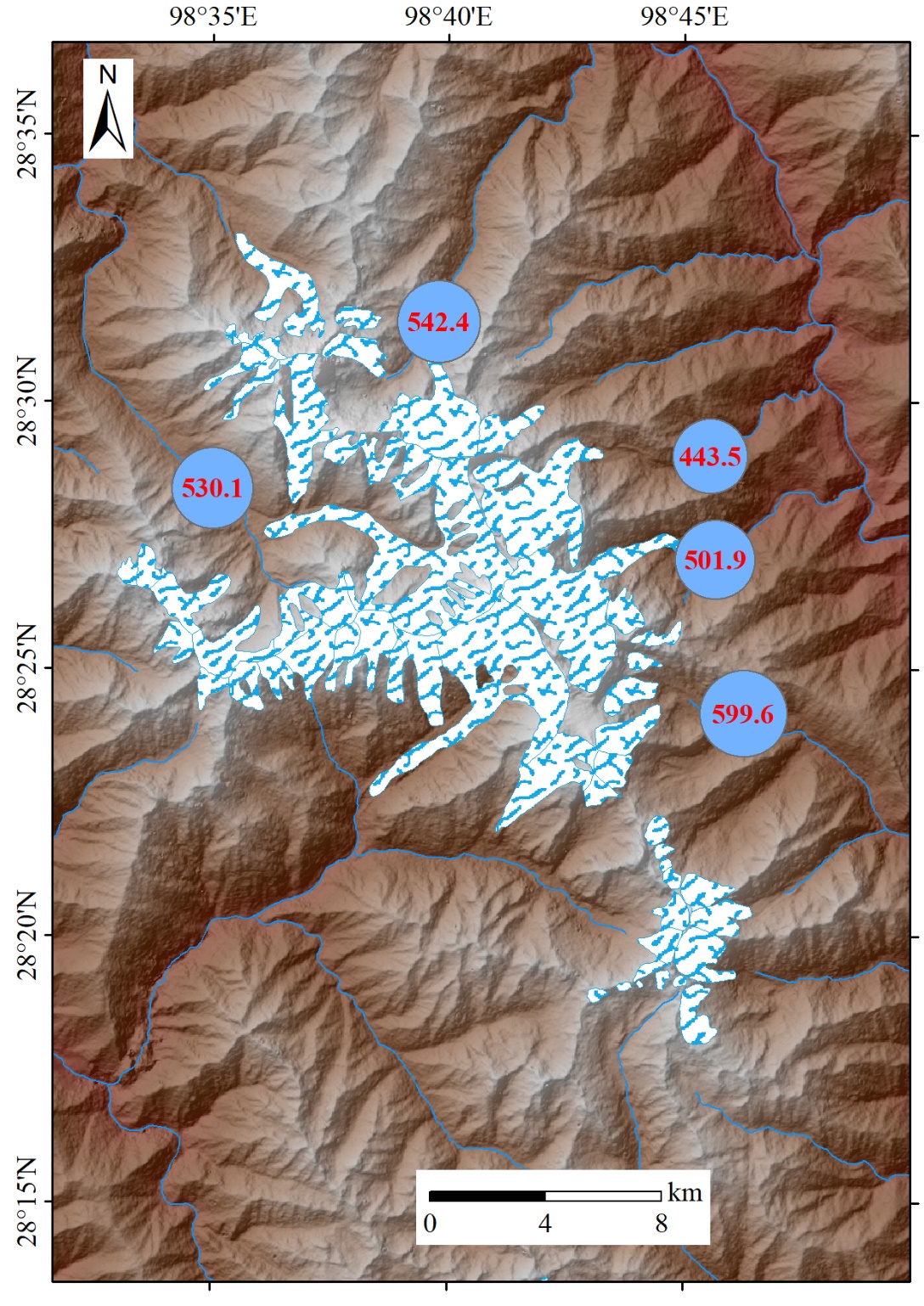


S1 Fig. Mean ∑PAHs (ng‧L^−1^) distribution in samples from the different watersheds. Data sources: Rivers and glaciers are extracted from Landsat 8 imagery, DEM (elevation) based on Advanced Spaceborne Thermal Emission and Reflection Radiometer (ASTER), was obtained from NASA (https://www.earthdata.nasa.gov/). All sources are in the public domain and not copyrighted.
