# Supplementary material for: Unveiling hidden threats: Polycyclic aromatic hydrocarbons pollution in the glacial waters of the Meili Snow Mountains in the southeastern Tibetan Plateau
Source: PLoS One. 2025 Oct 16;20(10):e0334592. doi: 10.1371/journal.pone.0334592 (PMC12530526; doi:10.1371/journal.pone.0334592)
Supplement: S1 Table — (DOCX) [file pone.0334592.s002.docx]

S1 Table. Detailed information of water samples collected from Meili Snow Mountains in the southeastern Tibetan Plateau

| Watershed | No. | Latitude(N) | Longitude(E) | Altitude(m) | Type |
| --- | --- | --- | --- | --- | --- |
| Qunatong River | gs-1 | 28.46066 | 98.59577 | 3829 | Glacier meltwater |
|  | gs-2 | 28.46090 | 98.59502 | 3847.2 | Glacier meltwater |
|  | gs-3 | 28.46613 | 98.61479 | 3936.1 | Glacier meltwater |
|  | gs-4 | 28.46135 | 98.60872 | 3898.7 | Glacier meltwater |
|  | gs-5 | 28.48100 | 98.57800 | 3763.3 | Glacier meltwater |
|  | gs-6 | 28.48593 | 98.56980 | 3749.9 | River water |
| Pojun River | pj-1 | 28.51964 | 98.65910 | 3985.6 | Glacier meltwater |
|  | pj-2 | 28.51971 | 98.65840 | 3992.7 | Glacier meltwater |
|  | pj-3 | 28.52813 | 98.64638 | 4541.5 | Glacier meltwater |
|  | pj-4 | 28.59302 | 98.70870 | 2919.9 | River water |
| Mingyong River | my-1 | 28.45306 | 98.75836 | 2818.2 | Glacier meltwater |
|  | my-2 | 28.46013 | 98.77366 | 2549 | River water |
| Sinong River | sn-1 | 28.47866 | 98.73947 | 3431.9 | Glacier meltwater |
|  | sn-2 | 28.47922 | 98.73892 | 3443.8 | Glacier meltwater |
|  | sn-3 | 28.48909 | 98.7709 | 2678.5 | River water |
| Yubeng River | yb-1 | 28.40846 | 98.74364 | 3882.8 | Glacier meltwater |
|  | yb-2 | 28.40903 | 98.74351 | 3860.7 | Glacier meltwater |
|  | yb-3 | 28.39987 | 98.77483 | 3242.9 | River water |
