# Supplementary material for: Unveiling hidden threats: Polycyclic aromatic hydrocarbons pollution in the glacial waters of the Meili Snow Mountains in the southeastern Tibetan Plateau
Source: PLoS One. 2025 Oct 16;20(10):e0334592. doi: 10.1371/journal.pone.0334592 (PMC12530526; doi:10.1371/journal.pone.0334592)
Supplement: S2 Table — (DOCX) [file pone.0334592.s003.docx]

S2 Table. Details of the recovery and precision of PAH detection

| Category | Detection times | Recovery (%) | Standard deviation (%) |
| --- | --- | --- | --- |
| Nap | 6 | 79.8 | 1.97 |
| Acy | 6 | 78.4 | 1.80 |
| Ace | 6 | 81.5 | 3.46 |
| Flu | 6 | 78.8 | 4.67 |
| Phe | 6 | 82.6 | 2.63 |
| Ant | 6 | 89.3 | 3.18 |
| Fluo | 6 | 86.2 | 3.73 |
| Pyr | 6 | 86.3 | 1.03 |
| BaA | 6 | 91.4 | 1.16 |
| Chry | 6 | 92.5 | 4.22 |
| BbF | 6 | 90.4 | 1.08 |
| BkF | 6 | 94.3 | 3.21 |
| BaP | 6 | 88.7 | 4.61 |
| IcdP | 6 | 92.2 | 4.63 |
| DahA | 6 | 93.4 | 3.12 |
| BghiP | 6 | 89.9 | 1.83 |
