# Supplementary material for: Unveiling hidden threats: Polycyclic aromatic hydrocarbons pollution in the glacial waters of the Meili Snow Mountains in the southeastern Tibetan Plateau
Source: PLoS One. 2025 Oct 16;20(10):e0334592. doi: 10.1371/journal.pone.0334592 (PMC12530526; doi:10.1371/journal.pone.0334592)
Supplement: S3 Table — (DOCX) [file pone.0334592.s004.docx]

S3 Table. TEF values of the 16 PAHs [1-3]

| Category | TEF | Category | TEF |
| --- | --- | --- | --- |
| Nap | 0.001 | BaA | 0.1 |
| Acy | 0.001 | Chry | 0.01 |
| Ace | 0.001 | BbF | 0.1 |
| Flu | 0.001 | BkF | 0.1 |
| Phe | 0.001 | BaP | 1 |
| Ant | 0.01 | IcdP | 0.1 |
| Fluo | 0.001 | DahA | 1 |
| Pyr | 0.001 | BghiP | 0.01 |

**References**

1. Environmental Protection Agency US. Guidelines for the health risk assessment of chemical mixtures. Federal Register 51, 34014-34025, 1986.
2. Cao Z, Liu J, Luan Y, Li Y, Ma M, Xu J, et al. Distribution and ecosystem risk assessment of polycyclic aromatic hydrocarbons in the Luan River, China. Ecotoxicology. 2010; 19: 827-837. doi: 10.1007/s10646-010-0464-5.
3. Liu X. Distribution and health risk assessment of polycyclic aromatic hydrocarbons and its derivatives in drinking water. M.Sc. Thesis, Southwest University. 2011.
