# Supplementary material for: Unveiling hidden threats: Polycyclic aromatic hydrocarbons pollution in the glacial waters of the Meili Snow Mountains in the southeastern Tibetan Plateau
Source: PLoS One. 2025 Oct 16;20(10):e0334592. doi: 10.1371/journal.pone.0334592 (PMC12530526; doi:10.1371/journal.pone.0334592)
Supplement: S4 Table — (DOCX) [file pone.0334592.s005.docx]

S4 Table. Key exposure parameters for health risk assessment [1,2]

| Parameter | Meaning | Unit | Infants | Toddlers | Children | Adolescents | Adults |
| --- | --- | --- | --- | --- | --- | --- | --- |
| IR | Water intake rate | L‧day^−1^ | 0.911 | 0.861 | 1.28 | 1.414 | 1.85 |
| EF | Exposure frequency | Days‧year^−1^ | 365 | | | | |
| ED | Exposure duration | Years |  | | | | |
| BW | Body weight | Kg | 11.2 | 19.6 | 36.8 | 54.8 | 60.6 |
| AT | Average lifespan | Days | 70 × 365 = 25550 | | | | |

**References**

1. Zhao XG, Duan XL. Exposure Factors Handbook of Chinese Population. Beijing: China Environmental Science Press; 2013.
2. Han XY. Distribution Characteristics and Risk Assessment of Typical Persistent Organic Pollutants in the Beijiang River of Qingyuan Section. M.Sc. Thesis, Hebei Normal University. 2018.
