# Supplementary material for: Unveiling hidden threats: Polycyclic aromatic hydrocarbons pollution in the glacial waters of the Meili Snow Mountains in the southeastern Tibetan Plateau
Source: PLoS One. 2025 Oct 16;20(10):e0334592. doi: 10.1371/journal.pone.0334592 (PMC12530526; doi:10.1371/journal.pone.0334592)
Supplement: S5 Table — (DOCX) [file pone.0334592.s006.docx]

S5 Table. C_NCs_ and C_MPCs_ values of the 16 PAHs [1]

| Category | C_NCs_ (ng‧L^−1^) | C_MPCs_ (ng‧L^−1^) | Category | C_NCs_ (ng‧L^−1^) | C_MPCs_ (ng‧L^−1^) |
| --- | --- | --- | --- | --- | --- |
| Nap | 12 | 1200 | BaA | 0.1 | 10 |
| Acy | 0.7 | 70 | Chry | 3.4 | 340 |
| Ace | 0.7 | 70 | BbF | 0.1 | 10 |
| Flu | 0.7 | 70 | BkF | 0.4 | 40 |
| Phe | 3 | 300 | BaP | 0.5 | 50 |
| Ant | 0.7 | 70 | IcdP | 0.5 | 50 |
| Fluo | 3 | 300 | DahA | 0.4 | 40 |
| Pyr | 0.7 | 70 | BghiP | 0.3 | 30 |

**References**

1. Aziz F, Syed JH, Malik RN, Katsoyiannis A, Mahmood A, Li J, et al. Occurrence of polycyclic aromatic hydrocarbons in the Soan River, Pakistan: insights into distribution, composition, sources and ecological risk assessment. Ecotoxicol Environ Saf. 2014; 109:77-84. doi:10.1016/j.ecoenv.2014.07.022.
