# Supplementary material for: Unveiling hidden threats: Polycyclic aromatic hydrocarbons pollution in the glacial waters of the Meili Snow Mountains in the southeastern Tibetan Plateau
Source: PLoS One. 2025 Oct 16;20(10):e0334592. doi: 10.1371/journal.pone.0334592 (PMC12530526; doi:10.1371/journal.pone.0334592)
Supplement: S6 Table — (DOCX) [file pone.0334592.s007.docx]

S6 Table. Risk levels of the individual PAHs and ∑PAHs [1]

| Individual PAHs | | | ∑PAHs | | |
| --- | --- | --- | --- | --- | --- |
| Risk level | RQ_NCs_ | RQ_MPCs_ | Risk level | RQ_NCs_ | RQ_MPCs_ |
| Risk free | 0 |  | Risk free | 0 | 0 |
|  |  |  | Low risk | ≥ 1, < 800 |  |
| Moderate risk | ≥ 1 | < 1 | Moderate risk −1 | ≥ 800 | 0 |
|  |  |  | Moderate risk −2 | < 800 | > 1 |
| High risk |  | ≥ 1 | High risk | ≥ 800 | ≥ 1 |

**References**

1. Liu F, Liu J, Chen Q, Wang B, Cao, Z. Pollution characteristics, ecological risk and sources of polycyclic aromatic hydrocarbons (PAHs) in surface sediment from Tuhai-Majia River system, China. Procedia Environ. Sci. 2012; 13: 1301-1314. doi: 10.1016/j.proenv.2012.01.123.
