# Supplementary material for: Unveiling hidden threats: Polycyclic aromatic hydrocarbons pollution in the glacial waters of the Meili Snow Mountains in the southeastern Tibetan Plateau
Source: PLoS One. 2025 Oct 16;20(10):e0334592. doi: 10.1371/journal.pone.0334592 (PMC12530526; doi:10.1371/journal.pone.0334592)
Supplement: S7 Table — (DOCX) [file pone.0334592.s008.docx]

S7 Table. Concentrations of individual PAHs and ∑PAHs (ng‧L^−1^) at each sampling point in the Meili Snow Mountains, southeastern Tibetan Plateau

| No. | Nap | Acy | Ace | Flu | Phe | Ant | Fluo | Pyr | BaA | Chry | BbF | BkF | BaP | IcdP | DahA | BghiP | ∑PAHs |
| --- | --- | --- | --- | --- | --- | --- | --- | --- | --- | --- | --- | --- | --- | --- | --- | --- | --- |
| gs-1 | <11.0 | <10.0 | <20.0 | 235 | 201 | <15.0 | <15.0 | 59.8 | <15.0 | <15.0 | <15.0 | <15.0 | 88.8 | <15.0 | <15.0 | <15.0 | 584.6 |
| gs-2 | <11.0 | <10.0 | <20.0 | 231 | 129 | <15.0 | <15.0 | 55.5 | <15.0 | <15.0 | <15.0 | <15.0 | 84.8 | <15.0 | <15.0 | <15.0 | 500.3 |
| gs-3 | <11.0 | <10.0 | <20.0 | 255 | 158 | <15.0 | <15.0 | 45 | <15.0 | <15.0 | <15.0 | <15.0 | 75.1 | <15.0 | <15.0 | <15.0 | 533.1 |
| gs-4 | <11.0 | <10.0 | <20.0 | 199 | 154 | <15.0 | <15.0 | 36 | <15.0 | <15.0 | <15.0 | <15.0 | 66.8 | <15.0 | <15.0 | <15.0 | 455.8 |
| gs-5 | <11.0 | <10.0 | <20.0 | 237 | 134 | <15.0 | <15.0 | 41.2 | <15.0 | <15.0 | <15.0 | <15.0 | 71.5 | <15.0 | <15.0 | <15.0 | 483.7 |
| gs-6 | <11.0 | <10.0 | <20.0 | 391 | 129 | <15.0 | <15.0 | 36.3 | <15.0 | <15.0 | <15.0 | <15.0 | 67 | <15.0 | <15.0 | <15.0 | 623.3 |
| pj-1 | <11.0 | <10.0 | <20.0 | 164 | 84.4 | <15.0 | <15.0 | 64.7 | <15.0 | <15.0 | <15.0 | <15.0 | 93.4 | <15.0 | <15.0 | <15.0 | 406.5 |
| pj-2 | <11.0 | <10.0 | <20.0 | 330 | 88.1 | <15.0 | <15.0 | 53.5 | <15.0 | <15.0 | <15.0 | <15.0 | 83 | <15.0 | <15.0 | <15.0 | 554.6 |
| pj-3 | <11.0 | <10.0 | <20.0 | 291 | 163 | <15.0 | <15.0 | 65.3 | <15.0 | <15.0 | <15.0 | <15.0 | 93.9 | <15.0 | <15.0 | <15.0 | 613.2 |
| pj-4 | <11.0 | <10.0 | 22.8 | 392 | 88.4 | <15.0 | <15.0 | 30.5 | <15.0 | <15.0 | <15.0 | <15.0 | 61.6 | <15.0 | <15.0 | <15.0 | 595.3 |
| my-1 | <11.0 | <10.0 | <20.0 | 324 | 113 | <15.0 | <15.0 | 39.9 | <15.0 | <15.0 | <15.0 | <15.0 | 70.4 | <15.0 | <15.0 | <15.0 | 547.3 |
| my-2 | <11.0 | <10.0 | <20.0 | 250 | 88.5 | <15.0 | <15.0 | 44 | <15.0 | <15.0 | <15.0 | <15.0 | 74.1 | <15.0 | <15.0 | <15.0 | 456.6 |
| sn-1 | <11.0 | <10.0 | <20.0 | 215 | 33 | <15.0 | <15.0 | 67 | <15.0 | <15.0 | <15.0 | <15.0 | 95.5 | <15.0 | <15.0 | <15.0 | 410.5 |
| sn-2 | <11.0 | <10.0 | <20.0 | 166 | 166 | <15.0 | <15.0 | 62.6 | <15.0 | <15.0 | <15.0 | <15.0 | 91.5 | <15.0 | <15.0 | <15.0 | 486.1 |
| sn-3 | <11.0 | <10.0 | <20.0 | 270 | 23.6 | <15.0 | <15.0 | 55.5 | <15.0 | <15.0 | <15.0 | <15.0 | 84.8 | <15.0 | <15.0 | <15.0 | 433.9 |
| yb-1 | <11.0 | <10.0 | <20.0 | 305 | 56.1 | <15.0 | <15.0 | 17.9 | <15.0 | <15.0 | <15.0 | <15.0 | 50 | <15.0 | <15.0 | <15.0 | 429 |
| yb-2 | <11.0 | <10.0 | <20.0 | 389 | 58.8 | <15.0 | <15.0 | 35.2 | <15.0 | <15.0 | <15.0 | <15.0 | 66 | <15.0 | <15.0 | <15.0 | 549 |
| yb-3 | <11.0 | <10.0 | 20.8 | 425 | 168 | <15.0 | <15.0 | 90.1 | <15.0 | <15.0 | <15.0 | <15.0 | 117 | <15.0 | <15.0 | <15.0 | 820.9 |
