# Supplementary material for: Unveiling hidden threats: Polycyclic aromatic hydrocarbons pollution in the glacial waters of the Meili Snow Mountains in the southeastern Tibetan Plateau
Source: PLoS One. 2025 Oct 16;20(10):e0334592. doi: 10.1371/journal.pone.0334592 (PMC12530526; doi:10.1371/journal.pone.0334592)
Supplement: S8 Table — (DOCX) [file pone.0334592.s009.docx]

S8 Table. Mean ∑PAHs (ng‧L^−1^) in water samples from different basins around the world

| Sample Region | Abbr. Sample Region | Sample Time | Sample Type | Mean ∑PAHs | Range | Ref. |
| --- | --- | --- | --- | --- | --- | --- |
| Baersi Snow Mountains | BES | 2022 | Glacier meltwater | 62.0 | – | [1] |
| Dagu Glacier | DG | 2022 | Glacier meltwater | 922.7 | 486–1380 | [1] |
| Daxia River | DXR | 2022 | River water | 1176.6 | 1120–1233.1 | [1] |
| Downstream of Hailuogou Glacier (Dadu River) | HGR | 2022 | River water | 246 | 177–315 | [1] |
| Hailuogou Glacier | HG | 2022 | Glacier meltwater | 28.5 | 18–39 | [1] |
| Buriganga and Dhaleswari Rivers | BDR | 2022 | River water | 5799.2 | 878.7–26086.8 | [2] |
| Cauca River | CR | 2010-2011 | River water | 2344.5 | 52.1–12888.2 | [3] |
| Chenab River | CR | 2007-2009 | River water | 629.5 | 289.64–994.88 | [4] |
| Coastal freshwater wetland | CFW | 2020 | River water | 78.3 | 5.14–253.37 | [5] |
| Coastal seawater Jeddah Coast | CSJC | 2021 | Coastal seawater | 360 | 151–748 | [6] |
| Ganga River | GR | 2022 | River water | 695.8 | 199.29–1587.6 | [7] |
| Gurenhekou Glacier | GRHK | 2008 | Snow | 24.6 | – | [8] |
| Qiyi Glacier | QG | 2008 | Snow | 31.2 | – | [8] |
| Xiaodongkemadi Glacier | XDKMD | 2008 | Snow | 20.5 | – | [8] |
| Yuzhufeng Glacier | YZF | 2008 | Snow | 60.6 | – | [8] |
| Hangzhou Coastal Bay | HCB | 2020 | Coastal seawater | 220 | 98.9–510 | [9] |
| Hangzhou Outer Bay | HOB | 2019 | Seawater | 130 | 76.8–198 | [9] |
| Headwater region of the Dongjiang River | HRDR | 2015-2016 | River water | 181.5 | 102.2–407.6 | [10] |
| Ice core of Dasuopu glaciers | ICDG | 2005 | Glacier | 26 | – | [11] |
| Ice core of East Rongbuk Glacier | ICRG | 2005 | Glacier | 100 | – | [12] |
| Lanhuagou Epikarst Springs | LES | 2019 | Springs water | 136.7 | – | [13] |
| Liaohe River | LR | 2011 | River water | 2920.6 | 840.5–4274.73 | [14] |
| Lower reaches of the Yangtze River | LRYR | 2019 | River water | 78.3 | 37.27–285.88 | [15] |
| Mississippi River | MR | 2004 | River water | 114.9 | 62.9–144.7 | [16] |
| Moscow River | MSR | 2013 | River water | 75.8 | 50.6–120.1 | [17] |
| Nam Co lake | NCL | 2014 | lake water | 28.8 | ND–84 | [18] |
| Ovia River | OR | 2012 | River water | 17560 | 2330–25830 | [19] |
| Pearl River Delta | PRD | 2005-2006 | River water | 164.0 | 21.8–497 | [20] |
| Qingdao Bays Coastal Seawater | QDBCS | 2021 | Coastal seawater | 399.7 | 120–614 | [21] |
| River Nile | RN | 2015-2016 | River water | 4332 | 235.92–10367.6 | [22] |
| River system of Tianjin | RST | 2006 | River water | 174 | 45.8–1272 | [23] |
| Samsun coastal seawater | SCS | 2019-2020 | Coastal seawater | 138.9 | 24.86–477.78 | [24] |
| Sergipe State River | SSR | 2022 | River water | 1856 | 0–7070 | [25] |
| Songhua River | SR | 2007 | River water | 910.5 | 152.95–2444.54 | [26] |
| Source of the Qiantang River | SQR | 2012 | River water | 79.8 | 2.42–303.35 | [27] |
| Southern Bohai Sea | SBS | 2018 | Coastal seawater | 35.3 | 26.9–50.1 | [28] |
| Surface water of Shanghai | SWS | 2012 | River water | 127.4 | 7.29–400.51 | [29] |
| Surface water of Kongsfjorden | SWK | 2016-2017 | Coastal seawater | 17.3 | 2.9–40.4 | [30] |
| Surface waters of the Ganga River | SWGR | 2019 | River water | 24750.0 | 1640–93000 | [31] |
| The Pacific sector of the Arctic | PA | 2018 | Seawater | 105.0 | 34.69–338.28 | [32] |
| The upper part of the Ganges River | UPGR | 2013 | River water | 31.7 | 13.9–43.3 | [33] |
| Typical drinking water sources in Wuhan | TDWW | 2020 | River water | 173.9 | 57.04–475.79 | [34] |
| Western shore of Admiralty Bay | WSAB | 2016 | Glacier meltwater | 224.7 | 0.2–1365 | [35] |
| Yangtze River | YR | 2021 | River water | 123.9 | 40.9–334.7 | [36] |
| Yarlung Tsangpo River | YTR | 2016 | River water | 465.3 | 315.4–670.1 | [37] |
| Yellow River | HR | 2005-2006 | River water | 662.0 | 144–2361 | [39] |
| Meili Snow Mountains | MLSM | 2023 | Glacier meltwater | 526.9 | 406.5–820.9 | This study |
| – indicates no relevant data. | | | | | | |

**References**

1. Liu X, Dong Z, Baccolo G, Gao W, Li Q, Wei T, et al. Distribution, composition and risk assessment of PAHs and PCBs in cryospheric watersheds of the eastern Tibetan Plateau. Sci Total Environ. 2023; 890: 164234. doi: 10.1016/j.scitotenv.2023.164234.
2. Nahar A, Akbor MA, Sarker S, Bakar Siddique MA, Shaikh MAA, Chowdhury NJ, et al. Dissemination and risk assessment of polycyclic aromatic hydrocarbons (PAHs) in water and sediment of Buriganga and Dhaleswari rivers of Dhaka, Bangladesh. Heliyon. 2023; 9: e18465. doi: 10.1016/j.heliyon.2023.e18465.
3. Sarria-Villa R, Ocampo-Duque W, Páez M, Schuhmacher M. Presence of PAHs in water and sediments of the Colombian Cauca River during heavy rain episodes, and implications for risk assessment. Sci Total Environ. 2016; 540: 455-465. doi: 10.1016/j.scitotenv.2015.07.020.
4. Farooq S, Eqani SA, Malik RN, Katsoyiannis A, Zhang G, Zhang Y, et al. Occurrence, finger printing and ecological risk assessment of polycyclic aromatic hydrocarbons (PAHs) in the Chenab River, Pakistan. J Environ Monit. 2011; 13(11): 3207-3215. doi: 10.1039/c1em10421g.
5. Cheshmvahm H, Keshavarzi B, Moore F, Zarei M, Esmaeili HR, Hooda PS. Investigation of the concentration, origin and health effects of PAHs in the Anzali wetland: The most important coastal freshwater wetland of Iran. Mar Pollut Bull. 2023; 193: 115191. doi: 10.1016/ j.marpolbul.2023.115191.
6. El-Maradny A, Orif MI, AlKobati A, Ghandourah MA, Al-Farawati RK. Polycyclic aromatic hydrocarbons in the water column of three hot spot areas, Jeddah coast, eastern of Red Sea. Reg Stud Mar Sci. 2023; 64: 103047. doi: 10.1016/j.rsma.2023.103047.
7. Naaz N, Pandey J. Spatial distribution of polycyclic aromatic hydrocarbons in water and sediment in the Ganga River: source diagnostics and health risk assessment on dietary exposure through a common carp fish Labeo rohita. Environ Geochem Health. 2024; 46(6): 196. doi: 10.1007/s10653-024-01980-x
8. Li Q, Wang N, Wu X, Pu J, He J, Zhang C. Sources and distribution of polycyclic aromatic hydrocarbons of different glaciers over the Tibetan Plateau. Sci China-Earth Sci. 2011; 54: 1189-1198. doi: 10.1007/s11430-010-4047-3.
9. Wu Y, Zhang Z, Huang W, Liu H, Zhang R, Jiao H, et al. Environmental profile, potential sources, and ecological risk of polycyclic aromatic hydrocarbons in a typical coastal bay and outer bay area. Environ Sci Pollut Res. 2023; 30(31):77757-77770. doi:10.1007/s11356-023-27885-3.
10. Chen Y, Zhang X, Zhang H, Cui K, Li F, Hassan M, et al. Distribution, Source Identification, and Risk Assessment of Polycyclic Aromatic Hydrocarbons in a Large Drinking Water River-Reservoir System. Water Air Soil Pollut. 2024; 235: 183. doi: 10.1007/s11270-024-06986-w.
11. Wang X, Yao T, Wang P, Yang W, Tian L. The recent deposition of persistent organic pollutants and mercury to the Dasuopu glacier, Mt. Xixiabangma, central Himalayas. Sci Total Environ. 2008; 394: 134-143. doi: 10.1016/j.scitotenv.2008.01.016.
12. Wang X, Xu B, Kang S, Cong Z, Yao T. The historical residue trends of DDT, hexachlorocyclohexanes and polycyclic aromatic hydrocarbons in an ice core from Mt. Everest, central Himalayas, China. Atmos Environ. 2008; 42: 6699-6709. doi: 10.1016/ j.atmosenv.2008.04.035.
13. Qi X, Lan J, Sun Y, Wang S, Liu L, Wang J, et al. Linking PAHs concentration, risk to PAHs source shift in soil and water in epikarst spring systems, Southwest China. Ecotoxicol Environ Saf. 2023; 264:115465. doi:10.1016/j.ecoenv.2023.115465.
14. Wang H, Liu Z, Sun L, Wang Y, Luo Q, Wu H, et al. Characterization and Risk Assessment of Polycyclic Aromatic Hydrocarbons in Surface Water from Liaohe River, Northeast China. Polycycl Aromat Compd. 2018; 38: 389-401. doi: 10.1080/10406638.2016.1220960.
15. Jia TQ, Lei RR, Wu XL, Ni TT, Sun SR, Guo W, Liu WB. [Distribution, Sources, and Risk Assessment of Polycyclic Aromatic Hydrocarbons (PAHs) in Tributary Waters of the Lower Reaches of the Yangtze River, China]. Huan Jing Ke Xue. 2020; 41(5):2221-2228. Chinese.
16. Zhang S, Zhang Q, Darisaw S, Ehie O, Wang, G. Simultaneous quantification of polycyclic aromatic hydrocarbons (PAHs), polychlorinated biphenyls (PCBs), and pharmaceuticals and personal care products (PPCPs) in Mississippi river water, in New Orleans, Louisiana, USA. Chemosphere. 2007; 66(6): 1057-69. doi: 10.1016/j.chemosphere.2006.06.067.
17. Eremina N, Paschke A, Mazlova EA, Schüürmann G. Distribution of polychlorinated biphenyls, phthalic acid esters, polycyclic aromatic hydrocarbons and organochlorine substances in the Moscow River, Russia. Environ Pollut. 2016; 210: 409-18. doi: 10.1016/j.envpol.2015.11.034.
18. Ren J, Wang X, Wang C, Gong P, Yao T. Atmospheric processes of organic pollutants over a remote lake on the central Tibetan Plateau: implications for regional cycling. Atmos Chem Phys. 2017; 17: 1401-1415. doi: 10.5194/acp-17-1401-2017.
19. Tongo I, Ezemonye L, Akpeh K. Distribution, characterization, and human health risk assessment of polycyclic aromatic hydrocarbons (PAHs) in Ovia River, Southern Nigeria. Environ Monit Assess. 2017; 189(6): 247. doi: 10.1007/s10661-017-5931-5.
20. Wang JZ, Nie YF, Luo XL, Zeng EY. Occurrence and phase distribution of polycyclic aromatic hydrocarbons in riverine runoff of the Pearl River Delta, China. Mar Pollut Bull. 2008; 57(6-12): 767-74. doi: 10.1016/j.marpolbul.2008.01.007.
21. Lu J, Li M, Tan J, He M, Wu H, Kang Y, et al. Distribution, sources, ecological risk and microbial response of polycyclic aromatic hydrocarbons in Qingdao bays, China. Environ Pollut. 2023; 338:122687. doi:10.1016/j.envpol.2023.122687.
22. Haiba NSA. Polycyclic Aromatic Hydrocarbons (PAHs) in the River Nile, Egypt: Occurrence and Distribution. Polycycl Aromat Compd. 2019; 39: 425-433. doi:10.1080/10406638. 2017.1340314..
23. Shi Z, Tao S, Pan B, Fan W, He XC, Zuo Q, et al. Contamination of rivers in Tianjin, China by polycyclic aromatic hydrocarbons. Environ Pollut. 2005; 134(1): 97-111. doi: 10.1016/ j.envpol.2004.07.014..
24. Tepe Y, Aydın H, Ustaoğlu F, Taştekin Ö. Occurrence and characteristics of PAHs in coastal seawater off the city of Samsun on the Black Sea coast of Turkey. Reg. Stud. Mar. Sci. 2024; 74:103552. doi: 10.1016/j.rsma.2024.103552.
25. de Souza Bery CC, Dos Santos Gois AR, Silva BS, da Silva Soares L, Santos LGGV, Fonseca LC, et al. Polycyclic aromatic hydrocarbons in surface water of rivers in Sergipe State, Brazil: A comprehensive analysis of sources, spatial and temporal variation, and ecotoxicological risk. Mar Pollut Bull. 2024; 202: 116370. doi: 10.1016/j.marpolbul.2024.116370.
26. Ding J. Temporal and spatial distribution of PCBs, OCPs and PAHs in Songhua River. M.Sc. Thesis, Harbin Institute of Technology. 2008.
27. Yu J. Concentration and distribution of PCBs and PAHs in Water and sediments from the source of the Qiantang river, China. M.Sc. Thesis, Zhejiang University of Technology. 2013.
28. Cao Y, Wang J, Xin M, Wang B, Lin C. Spatial distribution and partition of polycyclic aromatic hydrocarbons (PAHs) in the water and sediment of the southern Bohai Sea: Yellow River and PAH property influences. Water Res. 2024; 248: 120873. doi: 10.1016/j.watres. 2023.120873.
29. Wang XP. Environmental behaviors and risk of polycyclic aromatic hydrocarbons and polychlorinated biphenyls in the surface water of Shanghai. M.Sc. Thesis, East China Normal University. 2017.
30. Ademollo N, Spataro F, Rauseo J, Pescatore T, Fattorini N, Valsecchi S, et al. Occurrence, distribution and pollution pattern of legacy and emerging organic pollutants in surface water of the Kongsfjorden (Svalbard, Norway): Environmental contamination, seasonal trend and climate change. Mar Pollut Bull. 2021; 163:111900. doi: 10.1016/j.marpolbul.2020.111900. .
31. Kumar B, Verma VK, Kumar S, Gargava, P. Polycyclic Aromatic Hydrocarbons in Surface Waters from India: Possible Sources and Risk Assessment. Polycycl Aromat Compd. 2024; 44: 3161-3177. doi: 10.1080/10406638.2023.2231600.
32. Ye, J. Occurrence, Sources and Fate of Polycyclic Aromatic Hydrocarbons and Polychlorinated Biphenyls in the Pacific Sector of the Arctic. M.Sc. Thesis, Shanghai Ocean University. 2021.
33. Sharma BM, Nizzetto L, Bharat GK, Tayal S, Melymuk L, Sáňka O, et al. Melting Himalayan glaciers contaminated by legacy atmospheric depositions are important sources of PCBs and high-molecular-weight PAHs for the Ganges floodplain during dry periods. Environ Pollut. 2015; 206: 588-96. doi: 10.1016/j.envpol.2015.08.012.
34. Zhang K, Fu Q, Tu X,Chang S, Fan Y, Sun X, et al. [Pollution Characteristics and Risk Assessment of Typical POPs in Typical Drinking Water Sources in Wuhan]. Huan Jing Ke Xue. 2021; **42**, 5836-5847. Chinese.
35. Szopińska M, Szumińska D, Bialik RJ, Dymerski T, Rosenberg E, Polkowska Ż. Determination of polycyclic aromatic hydrocarbons (PAHs) and other organic pollutants in freshwaters on the western shore of Admiralty Bay (King George Island, Maritime Antarctica). Environ Sci Pollut Res. 2019; 26(18): 18143-18161. doi: 10.1007/s11356-019-05045-w.
36. Shang N, Wang C, Kong J, Yu H, Li J, Hao W, et al. Dissolved polycyclic aromatic hydrocarbons (PAHs-d) in response to hydrology variation and anthropogenic activities in the Yangtze River, China. J Environ Manage. 2023; 326: 116673. doi: 10.1016/ j.jenvman.2022.116673.
37. Liu J, Lu G, Yang H, Dang T, Yan Z. Ecological impact assessment of 110 micropollutants in the Yarlung Tsangpo River on the Tibetan Plateau. J Environ Manage. 2020; 262:110291. doi: 10.1016/j.jenvman.2020.110291.
38. Sun JH, Wang GL, Chai Y, Zhang G, Li J, Feng J. Distribution of polycyclic aromatic hydrocarbons (PAHs) in Henan Reach of the Yellow River, Middle China. Ecotoxicol Environ Saf. 2009; 72(5):1614-24. doi: 10.1016/j.ecoenv.2008.05.010.
