# Supplementary material for: Unveiling hidden threats: Polycyclic aromatic hydrocarbons pollution in the glacial waters of the Meili Snow Mountains in the southeastern Tibetan Plateau
Source: PLoS One. 2025 Oct 16;20(10):e0334592. doi: 10.1371/journal.pone.0334592 (PMC12530526; doi:10.1371/journal.pone.0334592)
Supplement: Attachment — Submitted filename: Response to Academic Editor.docx [file pone.0334592.s011.docx]

Thank you for your thoughtful review of our manuscript. We sincerely appreciate the time and expertise you and the reviewers dedicated to evaluating our work. Your constructive suggestions have significantly strengthened the paper. We have carefully addressed all comments in the revised manuscript, with changes highlighted in **red text**.

**Point-by-Point Response to Comments:**

1. **Comment:** Please ensure that your manuscript meets PLOS ONE's style requirements, including those for file naming. The PLOS ONE style templates can be found at https://journals.plos.org/plosone/s/file?id=wjVg/PLOSOne_formatting _sample_main_body.pdf and https://journals.plos.org/plosone/s/file?id=ba62/ PLOSOne_formatting_sample_title_authors_affiliations.pdf

**Response:** We have made modifications in the submitted revised manuscript according to the relevant guidelines.

2. **Comment:** In your Methods section, please provide additional information regarding the permits you obtained for the work. Please ensure you have included the full name of the authority that approved the field site access and, if no permits were required, a brief statement explaining why.

**Response:** Thank you for pointing this out. No permits were needed for water sample collection, as the study utilized publicly accessible water bodies and adhered to local environmental guidelines.

3. **Comment:** Thank you for stating the following in the Acknowledgments Section of your manuscript:

This work was funded by the National Natural Science Foundation of China (41861015, U2202207) and the Natural Science Foundation of the Department of Education of Inner Mongolia (NJZY22285). We thank LetPub (www.letpub.com.cn) for its linguistic assistance durin

We note that you have provided funding information that is not currently declared in your Funding Statement. However, funding information should not appear in the Acknowledgments section or other areas of your manuscript. We will only publish funding information present in the Funding Statement section of the online submission form.

Please remove any funding-related text from the manuscript and let us know how you would like to update your Funding Statement.

**Response:** Thank you for pointing this out. We remove funding-related text from the acknowledgments section of the manuscript. Please update the Funding Statement for our manuscript to:

This work was funded by the National Natural Science Foundation of China (41861015, U2202207) and the Natural Science Foundation of the Department of Education of Inner Mongolia (NJZY22285). The funders had no role in the study design, data collection and analysis, decision to publish, or preparation of the manuscript.

4. **Comment:** We note that Figures 1, 7, 9, S1 in your submission contain [map/satellite] images which may be copyrighted. All PLOS content is published under the Creative Commons Attribution License (CC BY 4.0), which means that the manuscript, images, and Supporting Information files will be freely available online, and any third party is permitted to access, download, copy, distribute, and use these materials in any way, even commercially, with proper attribution. For these reasons, we cannot publish previously copyrighted maps or satellite images created using proprietary data, such as Google software (Google Maps, Street View, and Earth). For more information, see our copyright guidelines: http://journals.plos.org/plosone/s/licenses-and-copyright.

We require you to either (1) present written permission from the copyright holder to publish these figures specifically under the CC BY 4.0 license, or (2) remove the figures from your submission:

**Response:** Thank you for pointing this out. In the submitted revised manuscript, relevant images have all been supplemented with data descriptions.

Fig 1. Locations of the sampling points in the Meili Snow Mountains. Data sources: Rivers and glaciers are extracted from Landsat 8 imagery, Tibetan Plateau boundary was obtained from National Tibetan Plateau / Third Pole Environment Data Center (https://cstr.cn/18406.11. Geogra.tpdc.270099), DEM (elevation) based on Advanced Spaceborne Thermal Emission and Reflection Radiometer (ASTER), was obtained from NASA (https://www.earthdata.nasa.gov/). All sources are in the public domain and not copyrighted.

Fig 7. Distribution of mean ∑PAHs (ng**‧**L^−1^) in water samples from various watersheds: areas surrounding TP (a), areas surrounding China (b), and areas around the world (c) Detailed information is provided in S8 Table. Basemap satellite images was obtained from the World Imagery Esri layer under a CC BY license. Sources: Esri,Maxar, Earthstar Geographics, and the GIS User Community. Content is the intellectual property of Esri and is used herein with permission. Copyright © 2025 Esri and its licensors. All rights reserved.

Fig 9. Cluster analysis of 72 h back trajectories in the Meili Snow Mountains between October 2021 and October 2023. The colored lines represent different clusters. Data sources: National border information was obtained from Natural Earth (http://www.naturalearthdata.com/). All sources are in the public domain and not copyrighted.

S1 Fig. Mean ∑PAHs (ng‧L^−1^) distribution in samples from the different watersheds. Data sources: Rivers and glaciers are extracted from Landsat 8 imagery, DEM (elevation) based on Advanced Spaceborne Thermal Emission and Reflection Radiometer (ASTER), was obtained from NASA (https://www.earthdata.nasa.gov/). All sources are in the public domain and not copyrighted.

The satellite imagery base map used in Figure 7 is from ESRI, and it complies with the Terms of Use for static maps outlined on the webpage https://doc.arcgis.com/en/arcgis-online/reference/static-maps.htm. Please see the screenshot of the webpage below.

5. **Comment:** Please include captions for your Supporting Information files at the end of your manuscript, and update any in-text citations to match accordingly. Please see our Supporting Information guidelines for more information: http://journals.plos.org/plosone/s/supporting-information.

**Response:** Thank you for pointing this out. The captions for your Supporting Information files has been added to the submitted revised manuscript.

**Supporting information**

S1 Fig. Mean ∑PAHs (ng‧L^−1^) distribution in samples from the different watersheds. Data sources: Rivers and glaciers are extracted from Landsat 8 imagery, DEM (elevation) based on Advanced Spaceborne Thermal Emission and Reflection Radiometer (ASTER), was obtained from NASA (https://www.earthdata.nasa.gov/). All sources are in the public domain and not copyrighted. (DOCX)

S1 Table. Detailed information of water samples collected from Meili Snow Mountains in the southeastern Tibetan Plateau. (DOCX)

S2 Table. Details of the recovery and precision of PAH detection. (DOCX)

S3 Table. TEF values of the 16 PAHs. (DOCX)

S4 Table. Key exposure parameters for health risk assessment. (DOCX)

S5 Table. C_NCs_ and C_MPCs_ values of the 16 PAHs. (DOCX)

S6 Table. Risk levels of the individual PAHs and ∑PAHs. (DOCX)

S7 Table. Concentrations of individual PAHs and ∑PAHs (ng‧L^−1^) at each sampling point in the Meili Snow Mountains, southeastern Tibetan Plateau. (DOCX)

S8 Table. Mean ∑PAHs (ng‧L^−1^) in water samples from different basins around the world. (DOCX)
