# Supplementary material for: Unveiling hidden threats: Polycyclic aromatic hydrocarbons pollution in the glacial waters of the Meili Snow Mountains in the southeastern Tibetan Plateau
Source: PLoS One. 2025 Oct 16;20(10):e0334592. doi: 10.1371/journal.pone.0334592 (PMC12530526; doi:10.1371/journal.pone.0334592)
Supplement: Attachment — Submitted filename: Response to the Review Report ( Reviewer1 )2.docx [file pone.0334592.s012.docx]

Thank you for your thoughtful review of our manuscript. We sincerely appreciate the time and expertise you and the reviewers dedicated to evaluating our work. Your constructive suggestions have significantly strengthened the paper. We have carefully addressed all comments in the revised manuscript, with changes highlighted in **red text**.

**Point-by-Point Response to Comments:**

1. **Comment:** The revised manuscript states that there has been no reported research on POPs in the Meili Snow Mountains to date. However, the authors have already published a research paper on polychlorinated biphenyls (PCBs) in the Meili Snow Mountains this year. For details, please refer to: Zhang, Huawei, et al. "Glacial Waters Under Threat: Risk Assessment and Source Identification of Polychlorinated Biphenyls in Meili Snow Mountains, Southeastern Tibetan Plateau." Toxics 13.5 (2025): 391. It is recommended to amend the relevant statements and accurately summarize the current research status.

**Response:** We fully agree with this suggestion. We have added relevant content and reference (lines 91-93;lines 625-627) in the submitted revised manuscript.

Zhang et al revealed that polychlorinated biphenyls contamination in the Meili Snow Mountains, predominantly from glacier melt and atmospheric transport, poses significant ecological risks but negligible carcinogenic threats to human populations [17].

17. Zhang H, Yao Y, Wen X, Zhang R, Liu R. Glacial Waters Under Threat: Risk Assessment and Source Identification of Polychlorinated Biphenyls in Meili Snow Mountains, Southeastern Tibetan Plateau. *Toxics*. 2025; 13: 391. doi: 10.3390/toxics13050391.

2. **Comment:** Some issues were responded to but have not been fully addressed. I understand that certain facts indeed cannot be changed; however, since the questions were raised, appropriate solutions should still be provided. For example:

The claim that PAHs are POPs.

The authors insist on using average concentrations for comparative analysis without providing statistical test results, which is inappropriate.

The reviewers pointed out the lack of description regarding the collection and analysis of environmental samples, which should be supplemented. However, the authors responded by stating that the number of available samples is limited, which seems unrelated to the question raised.

**Response:** We fully agree with this suggestion.

The claim that PAHs are POPs.

We have eliminated all descriptions of PAHs as POPs in both the Abstract and the main text.

Polycyclic aromatic hydrocarbons (PAHs) have posed considerable threats to both ecosystems and human health.

The environmental threats posed polycyclic aromatic hydrocarbons (PAHs), have attracted widespread attention due to public awareness of health problems increases.

The authors insist on using average concentrations for comparative analysis without providing statistical test results, which is inappropriate.

As recommended, we have incorporated a detailed description of the statistical analysis (lines 185-190) in the Materials and Methods section and added the corresponding analytical results (lines 245-249) to the Results section.

The concentrations of PAH components at the 18 sampling sites, which were distributed across five glacial watersheds, were statistically analyzed using Origin software. Since the data were not normally distributed, the non-parametric Mann-Whitney U test (for pairwise comparisons) and Kruskal-Wallis H test (for multi-group comparisons) were applied to evaluate the significance of differences in PAHs concentrations among the different watersheds.

The statistical analysis revealed significant differences (p < 0.05) in the concentrations of individual PAH components among the five glacial watersheds. Furthermore, a consistent spatial pattern was observed within each watershed, characterized by higher concentrations at downstream sites compared to upstream sites.

The reviewers pointed out the lack of description regarding the collection and analysis of environmental samples, which should be supplemented. However, the authors responded by stating that the number of available samples is limited, which seems unrelated to the question raised.

We have revised the manuscript by making the necessary modifications and additions (lines 104-120; lines 164-174).

Glacial river samples were collected from different river watersheds in the Meili Snow Mountains using a clean plastic bucket in October 2023. Sampling sites included the Qunatong River (gs), Pojun River (pj), Mingyong River (my), Sinong River (sn), and Yubeng River (yb) (Fig. 1). Five river water samples were collected from the downstream regions of rivers originating from glacial meltwater across various altitudinal gradients. Notably, the yb and gs watersheds are tourist destinations. In total, 18 water samples were collected at different altitudes along the glacier basins. Comprehensive details are provided in S1 Table.

During sampling, we employed the clean polypropylene suits, gloves, and a pre‒cleaned stainless steel shovel to prevent pollution and ensure the accuracy of subsequent laboratory measurements. In the field, water samples were filtered using 0.7 μm glass‒fiber filters (Whatman International Ltd., Maidstone, England). Samples of filtered water (2 L) was stored in low–density polyethylene bottles (Thermo Scientific), in the dark at 4℃ during transport to the analytical laboratory at the Beijing Institute of Geology of Nuclear Industry, where they were subsequently stored at −18℃ until analysis. Before sampling, these bottles were thoroughly rinsed with ultrapure water and acetone to remove potential organic pollutants.

A five‒point internal calibration curve was established for each of the 16 PAHs through serial dilution of a high‒concentration stock solution (200 mg**‧**L^−1^ of 16 PAH mixture in acetonitrile) to generate five calibration at concentrations of 0.1, 0.5, 1.0, 5.0, and 10.0 μg**‧**mL^−1^. A 10 μL aliquot of each calibration standard was automatically injected into the GC‒MS system using an autosampler to obtain chromatograms. Calibration curves were constructed by plotting the peak areas against corresponding concentrations, all of which exhibited excellent linearity (R² > 0.999). Quantification of individual PAHs was performed using their respective calibration curves. For MS detection, the scan mode was used a mass‒to‒charge (m/z) range of 35‒500. Speak identification of PAHs was performed using the National Institute of Standards and Technology (NIST) mass spectral library.
